# The effect of school exposure and personal contact on attitudes towards bullying and autism in schools: A cohort study with a control group

**DOI:** 10.1177/1362361320937088

**Published:** 2020-07-15

**Authors:** Anna Cook, Jane Ogden, Naomi Winstone

**Affiliations:** University of Surrey, UK

**Keywords:** adolescents, bullying, inclusion, neurodiversity, peer attitudes, school-age children, school climate, social exclusion, social identity

## Abstract

Research shows that the attitudes of children and adolescents towards bullying are influenced by the school environment and their peer groups. Given the increased vulnerability to bullying for autistic children, this study explored whether neurotypical children’s attitudes towards bullying and autism varied according to school exposure and personal contact with autistic people. Survey data were collected at the beginning and end of the school year from 775 children aged 11–12 years, from six schools: three with specialist centres for autism and three without. Participants read vignettes depicting bullying scenarios then completed measures of their attitudes in relation to the vignette and towards autism. Children from centre schools showed a greater increase in prosocial emotions towards bullying. For children from non-centre schools, an interaction showed a decrease in prosocial emotions except in response to social exclusion of an autistic child. Increases in personal contact showed a greater increase in positive attitudes towards autistic people. Explanations draw on theories of inter-group contact and social-moral reasoning. Results highlight the need for contact both at a personal level and through attending a school with an inclusive autism provision to increase understanding, improve attitudes towards autism and reduce tolerance for bullying.

**Lay abstract:**

Autistic children are more likely than non-autistic children to be bullied at school. This study therefore explored whether the kind of school setting and the level of personal contact with autistic people can affect children’s attitudes towards bullying and autism. Surveys were completed at the beginning and end of the school year by 775 children aged 11–12 years, from six schools: three with specialist centres for autistic children and three without. Participants read stories describing bullying situations, then provided their views in relation to the story and in relation to autism. Children in schools with centres increased their feelings of anger, pity, sadness and shame in response to the bullying situations. In contrast, children in schools with no centre showed less sociable responses to bullying, except in response to a story describing an autistic child, being excluded by classmates. Furthermore, children who increased the time they spent with autistic individuals over the course of the year showed a greater rise in positive attitudes towards autistic people. This highlights the need for both personal contact and an inclusive school environment, to improve attitudes towards autism and reduce tolerance for bullying.

## Introduction

Autistic children experience greater exposure to victimization and bullying (Bejerot & Mörtberg, 2009; Chatzitheochari et al., 2014; Humphrey & Symes, 2011; National Autistic Society (NAS), 2003; Symes & Humphrey, 2010). Various factors contribute to this, such as differences in social understanding (Garner & Hinton, 2010; Wainscot et al., 2008) and the negative beliefs and stereotypes held by neurotypical (NT) individuals leading to prejudice and discrimination (Humphrey & Symes, 2011). Research indicates that bullying of autistic children can have a multitude of outcomes including damaged self-esteem, mental health difficulties and higher rates of suicidal ideation (Bellini, 2004; Drahota, 2009; Hebron & Humphrey, 2012; Kim et al., 2000; Mayes et al., 2013; Reid & Batten, 2006; Rybczynski et al., 2018).

Many school-based programmes have been designed to prevent bullying. Systematic reviews and meta-analyses of whole school anti-bullying programmes report mixed results, with many finding little evidence of meaningful change (Baldry & Farrington, 2007; Ferguson et al., 2007; Merrell et al., 2008; J. D. Smith et al., 2004). An alternative intervention is to inform children about human differences, such as autism, with the goal of increasing acceptance of difference and celebrating diversity within the school context. However, studies again showed mixed findings with some reporting improved knowledge and attitudes after receiving descriptive and explanatory information (Campbell et al., 2004), but failure to influence behavioural intentions (Staniland & Byrne, 2013) and other research suggesting that information interventions are ineffective and even detrimental to intergroup attitudes since they highlight stereotypic activities (Bigler, 1999).

It is important to consider possible explanations for attitudes and behaviours during childhood and adolescence. Social domain theory (Turiel, 2008) suggests that there are changes in social and moral reasoning during adolescence. Whilst they are developing more advanced understanding about morality, they may become more likely to give priority to group functioning and social conventions, and be more willing to exclude members of outgroups based on these factors in certain intergroup contexts (Abrams et al., 2008; Abrams & Rutland, 2008; Killen & Rutland, 2011; Turiel, 2008). Abrams et al. (2008) found that older children (10–11 years) were more willing than younger children (5–7 years) to exclude peers on the basis of group membership, and the stronger children identified with their group, the more likely they were to exclude on this basis. These studies have implications for autistic children and their experiences of social exclusion (a form of indirect bullying, Olweus, 1993). Where NT children strongly identify with their ingroup, social domain theory suggests that decisions to exclude autistic peers may not be considered as ‘bullying’, but instead be justified as legitimate in order to protect group functioning, even when they understand moral implications.

One method found to be effective in facilitating more inclusive attitudes is through contact with members of other groups (Killen & Rutland, 2011). The Contact hypothesis (Allport, 1954) states that prejudice is a consequence of unfamiliarity and that contact can disconfirm negative stereotypes and instil more positive attitudes, beliefs and behaviours. Studies have found that across time, cross-group friendships predict positive outgroup evaluations (Feddes et al., 2009). Various studies have explored attitudes towards peers with special needs, finding that contact increased knowledge and led to more positive cognitive attitudes and behavioural intentions (Mavropoulou & Sideridis, 2014), inclusive attitudes (Grütter et al., 2017) and significantly more ‘liking’ (Maras & Brown, 1996). Furthermore, diagnostic disclosure and understanding of autism leads to more consistent social support at school (Hewstone & Brown, 1986; Ochs et al., 2001) and improved first impressions of autistic people by NT adults (Sasson & Morrison, 2019). Ochs et al. (2001) concluded that

It is unrealistic to expect that children without autism, rooted in biology and culture, can shed their self-consciousness and conventionality to imagine the world through autistic eyes. Yet, giving autism a greater dialogic space in the school curriculum may enhance the perspective-taking skills and nurture the creative potentialities of all children in inclusion classrooms. (p. 416)

Inclusive school settings provide opportunities for contact with peers who may exhibit unfamiliar characteristics. School settings can influence attitudes towards autism, particularly those that have established a school ethos celebrating diversity and accepting difference (e.g. Morewood et al., 2011). Morewood, Humphrey and Symes discussed the importance of focusing not only on the physical environment (e.g. the slopes of the ceilings and open-plan layouts) but also the social environment, such as giving safe and structured opportunities to interact with their peers (e.g. through supported activities and clubs). Likewise, the belief of teachers in the value of diversity is important, since this moderates the association between intergroup friendship and intentions for social exclusion (Grütter & Meyer, 2014). One example of a model of inclusion that attempts to meet these goals is the opening of three purpose-built specialist centres for autistic pupils in mainstream schools in England (NAS, 2015). These offer a unique opportunity to explore the impact of improved physical and social environments on changes in the attitudes of NT children towards their autistic peers.

This study therefore aimed to explore the impact of exposure to autism (a) through attending a school with a specialist centre for autistic pupils and (b) through personal contact with autistic people. Given the high levels of bullying of autistic children, the study focuses on the impact of these two types of exposure on the attitudes of NT children towards verbal bullying, the most commonly identified bullying violation (Ahmed & Smith, 1994; Boulton et al., 2002), and also towards social exclusion (in light of studies cited above highlighting the tendency for adolescents to exclude outgroup members to protect group functioning). While previous studies ask participants to assume the imagined role of the victim or bully, in this study participants were asked to imagine themselves watching the scene take place, that is, as a *bystander*, since this is the most likely role in bullying scenarios, and bystander responses have been found to have a significant influence on bullying scenarios (Salmivalli et al., 1996).

While most studies of bystander intentions place an emphasis on cognitive attitudes and reasoning, group-based emotions have also been found to impact behaviour in response to a transgression (Branscombe & Doosje, 2004; Brown et al., 2008). For example, studies show that strength of identification with a group can determine the experience and intensity of emotion and also behavioural intentions in response to bullying (Jones et al., 2009; Mackie et al., 1999). This study will therefore address affective responses in addition to judgements and behavioural intentions in response to the bullying of autistic children. Finally, the study will explore attitudes towards autism in general, to assess cognitive attitudinal differences according to contact.

The hypotheses are as follows:

1. *Hypothesis 1 (H1).* In comparison to NT children in schools without specialist centres for autistic pupils, NT children in schools with specialist centres for autistic pupils will show greater prosocial judgements, emotions and behavioural intentions towards bullying (both verbal bullying and social exclusion) (H1a); greater prosocial judgements, emotions and behavioural intentions when targets of bullying are autistic (H1b); and more positive attitudes towards autism (H1c).
2. *Hypothesis 2 (H2).* In comparison to NT children who decrease or have no change in their personal contact with autistic people, NT children who increase their personal contact with autistic people will show greater prosocial judgements, emotions and behavioural intentions towards bullying (both verbal bullying and social exclusion) (H2a); greater prosocial judgements, emotions and behavioural intentions when targets of bullying are autistic (H2b); and more positive attitudes towards autism (H2c).

## Method

### Design

The study adopted a factorial design, where the between-subject factors were (a) school exposure and (b) change in personal contact with autistic people. The dependent variables (DVs) were the degree of change over the course of one school year in (a) judgement of the treatment of a target (autistic or NT) in a bullying scenario (verbal bullying or social exclusion), (b) emotions in response to the same bullying scenario, (c) intended behaviours in response to the same bullying scenario and (d) attitudes towards autistic people.

### Participants

Participants were recruited from six urban mainstream secondary schools in South East England with broadly matched socioeconomic status (schools ranged from 8.6% to 18.5% eligibility for free school dinners compared to the country average of 27.7%). Three schools had specialist centres for autism and three schools had no specialist centres. Of 1050 participants recruited at baseline, 64 provided no data so were removed from the data set. 26 participants had a diagnosis of an autistic spectrum disorder (ASD), so were also removed for the purposes of this analysis. The sample at baseline therefore consisted of 960 participants (494 male; 466 female). At follow-up, 185 did not provide data (110 male; 75 female). The sample for the final analysis therefore consisted of 775 participants (391 female; 384 male). A power analysis was conducted using G*Power (Faul et al., 2007) that indicated with a small effect size of 0.1 (approximate average from the literature), significance of 0.05 and power of 0.80, a sample of 779 would be required. Recruitment from each school ranged from 109 to 174 participants, with centre schools providing a larger sample (*n* = 426) compared to non-centre schools (*n* = 349). There was no significant difference in the percentage of autistic pupils attending centre schools (*M* = 2.90%, *SD* = 0.01) and non-centre schools (*M* = 1.94%, *SD* = 0.005), *p* = 0.20. The mean age was 11.15 years (*SD* = 0.36 years). 643 participants were White, 49 Mixed-Race, 33 Asian, 7 Black and 43 Other/missing data. Comparisons of participant demographics by school exposure revealed no significant differences other than by ethnic background, with a higher percentage of White participants in centre schools (85.1%) than in non-centre schools (81.1%). This study received a favourable opinion by the University Ethics Committee (Ref: UEC/2016/051/FHMS).

### School exposure

#### Centre schools

These were mainstream secondary schools with purpose-built specialist centres, known as ‘Cullum Centres’, (NAS, 2015). The centres’ planning and design were consistent with research on how physical environments can affect autistic people. Natural light, ventilation, quiet areas and calm spaces were therefore integral to their design. Autistic pupils spend the majority of their lessons with their mainstream peers, enabling them to benefit from the greater opportunities afforded them by being a member of a mainstream school community. At the same time, the centres provide specialist support from trained staff and a calm setting to which pupils can retreat and/or develop their social or learning skills. Importantly, the schools also implement personal, social and health education (PSHE) programmes about autism, with the goal of further raising the salience of autism, reducing uncertainty and encouraging a positive inclusive school culture. While these schools do not provide higher exposure in terms of numbers of autistic pupils, they do provide higher exposure through making autism more ‘visible’ in the school and in terms of increased awareness, understanding and attention given to autism. Centre schools provided a median of 4 h of PSHE about autism/special educational needs (SEN)/disability/difference and diversity in Year 7.

#### Non-centre schools

These were mainstream secondary schools with no specialist centre for autistic pupils, but with regular SEN policy and provision for pupils with special education needs. Non-centre schools provided a median of 3 h of PSHE about autism/SEN/disability/difference and diversity in Year 7.

### Change in personal contact with autistic people

This variable was constructed by asking participants to rate the time they currently spend with autistic people using a Likert-type scale from ‘never’ to ‘very often’. A median split was used at each time point to categorize participants as either having low contact (below the median) or high contact (above the median). This resulted in three categories of personal contact:

Decrease: pupils with high contact at baseline and low contact at follow-up.
No change: pupils with high contact at baseline and high contact at follow-up or low contact at baseline and low contact at follow-up.
Increase: pupils with low contact at baseline and but high contact at follow-up.

### Procedure

Near the beginning of their school year, participants (Year 7 pupils, new to the school) were asked to complete baseline measures outlined below. An ‘opt-out’ consent procedure was employed, whereby parents were notified before the start of the study and could revoke consent for the participation of their child. Pupils could also choose not to participate on the day of testing. The study was conducted in school classrooms, with each class consisting of approximately 30 pupils and a teacher always present. Pupils were informed that the researcher was interested in finding out about their attitudes towards their peers. Before the questionnaire was completed, the researcher read aloud the instructions and emphasized that their answers would be anonymous. Questionnaires were paper-based and completed under controlled conditions.

### Vignettes

Judgements, emotions and intended behaviours were measured using vignettes developed in line with recommendations regarding length, neutrality, relevance and relatability (Evans et al., 2015; Hughes & Huby, 2004). The use of vignettes in previous studies was drawn upon and drafts were then scrutinized by co-authors for their sense, clarity, cultural neutrality and validity. Once finalized, the vignettes were subjected to a pilot testing process. Vignettes depicted a bullying scenario with an autistic *or* NT target experiencing either verbal bullying *or* social exclusion. For example, below is the vignette depicting an autistic girl experiencing social exclusion:

Emily is a girl in your year group. You don’t know her well, but have been told that she has autism – a brain condition that causes her to have difficulties communicating with other people and to get anxious and even angry when things change unexpectedly or when there is lots of noise. One day Emily walks up to you and some friends from your form and asks if she can join in your conversation. Amy – one of the girls in your group says ‘no, we’re having a private conversation’ and then turns her back on Emily to indicate that she should leave. This is not the first time it’s happened.

In the vignette with an NT target, the target was not identified as ‘neurotypical’, but was described as being self-conscious about their weight (vignettes available in Supplemental Material S1). Varying the target type (ASD/NT) and bullying type (verbal/social exclusion) enabled us to measure the impact of these additional variables on attitudes. Due to time restrictions precluding responses from participants to every vignette, participants were allocated quasi-randomly (i.e. in sequence according to where they sat in the room) to one of the four vignettes. Allocation of questionnaires to participants who completed both baseline and follow-up questionnaires was as follows: 120 pupils in centre schools and 98 in non-centre schools received vignette 1: NT target + verbal bullying; 106 in centre schools and 96 in non-centre schools received vignette 2: NT target + social exclusion; 107 in centre schools and 79 in non-centre schools received vignette 3: autistic target + verbal bullying; and 93 in centre schools and 76 in non-centre schools received vignette 4: autistic target + social exclusion.

Characters within the vignettes were the same gender as the participant. Some participants were assisted in vignette and questionnaire reading, so as not to exclude those with reading difficulties. Participants were given 20–30 min to complete the questionnaire.

Participants then completed the following:

(a) Demographics: participants were asked to provide demographic information including gender, age, disability/SEN, ethnic background and number of people they know/friends/family members on the autistic spectrum, and answer three identifier questions enabling baseline and follow-up questionnaires to be matched without the use of participant names.
(b) Personal contact: participants rated the time they currently spend with people they know who are autistic using a Likert-type scale from ‘never’ to ‘very often’.
(c) DVs:
  1. Judgements.
     This set of eight items related to judgements about what happened to the target, including four prosocial judgements (e.g. ‘How much do you think what happened to Emily/Jack was mean?’) and four antisocial judgements (e.g. ‘How much do you think what happened to Emily/Jack was funny?’). Participants indicated their agreement on five-point Likert scales, (1 = *not at all*; 5 = *extremely*). Responses to antisocial items were reverse coded. The maximum score was 40, but mean scores of the eight items were computed for analysis, giving a maximum mean score of 5. The scale had good internal consistency (α = 0.74).
  2. Emotions.
     This set of eight items related to their emotional response to the incident, where participants were asked, ‘How strongly do you think you would feel the following emotions . . .?’ with four prosocial items (e.g. ‘angry’, ‘sad’) and four antisocial items (e.g. ‘excited’, ‘satisfied’). Participants indicated their agreement on five-point Likert scales (1 = *not at all*; 5 = *extremely*). Responses to antisocial items were reverse coded. The maximum score was 40, but mean scores of the eight items were computed for analysis, giving a maximum mean score of 5. The scale had good internal consistency (α = 0.71).
  3. Intended behaviours.
     This set of eight items concerned their intended behaviours in response to the bullying scenario. Again there were four prosocial items (e.g. ‘How likely would you be to report it to a teacher?’, ‘How likely would you be to smile at Emily/Jack to show support for her/him?’) and four antisocial items (e.g. ‘How likely would be to do nothing?’, ‘How likely would you be to laugh’). Participants indicated their agreement on five-point Likert scales (1 = *definitely not*; 5 = *definitely*). Responses to antisocial items were reverse coded. The maximum score was 40, but mean scores of the eight items were computed for analysis, giving a maximum mean score of 5. The scale had good internal consistency (α = 0.72).
  4. Attitudes to autism.
     The Adjectives Checklist (ACL) (Siperstein, 1980) was used to measure attitudes towards people on the autistic spectrum. This scale is designed to mirror the behaviour of children in classroom settings where children express their opinions or beliefs about a peer using common descriptors such as ‘mean’ and ‘friendly’. Participants were asked to think of a person they know who is autistic and to circle words from a list of 34 adjectives (17 with positive valence, 17 with negative valence) describing a peer’s affective feelings, physical appearance, academic behaviour and social behaviour that they would use if they had to describe this person to their classmates. They were told they could use as many or as few words as they want. A composite score was calculated in which the number of negative adjectives chosen was subtracted from the number of positive adjectives chosen, and a construct of 20 was added. A resulting score below 20 represents a negative attitude toward autistic people and a score above 20 represents a positive attitude. The ACL has good construct validity and Cronbach’s alpha reported to range from 0.67 to 0.91. In this study, Cronbach’s alpha coefficient was 0.87.

### Data analysis

The data were analysed in the following ways:

(a) To screen data for missing values and normality.
(b) To explore the main effects of school exposure on changes in judgements, emotions, intended behaviours and attitudes towards autism using analysis of variance (ANOVA).
(c) To explore the main effects of personal contact with autism on changes in judgements, emotions, intended behaviours and attitudes towards autism using ANOVA.

### Community involvement

This study involved the autistic community at a number of levels. First, the research questions emerged from existing literature and previous interviews with autistic young people and their parents (Cook et al., 2016, 2017); second, these research questions were developed and formulated for this study in collaboration with a working group established by the National Autistic Society (NAS) to evaluate the effectiveness of the newly opened Cullum Centres. Third, NT and autistic children helped shape the research materials through a pilot process and gave their feedback on the vignettes and questions asked. Finally, interpretation of findings was discussed with head teachers of the centre schools and the NAS at the Cullum Centre leadership meeting and then disseminated through poster presentation at the Annual Meeting of the International Society for Autism Research.

## Results

### Data screening

Missing value analysis revealed no serious problems regarding patterns of missing data with the exception of the item ‘How often to you spend time these days with people that you know to have autism?’ For this item, there were 85 missing values (11%) out of a total of 775. As this was used as an independent variable, these participants were excluded from this part of the analysis. Little’s missing completely at random (MCAR) test confirmed that data were missing completely at random in relation to age, gender, ethnicity, school exposure, number of people they know/friends/family members on the autistic spectrum. For cases where one out of four prosocial or one out of four antisocial items was missing, the mean of the three supplied scores was imputed. Otherwise, pairwise deletion of missing data was implemented. Preliminary checks were conducted to ensure that there was no violation of assumptions of normality. None of the variables were skewed, but there were a number of variables with positive kurtosis. All except one variable (change in judgement by personal contact) showed homogeneity of variance. Alpha was set to 0.05.

### The impact of school exposure

#### DVs 1, 2 and 3: judgements, emotions and intended behaviours

To test the hypotheses that attending a school with a centre would lead to a greater increase in prosocial judgements, emotions and intended behaviours in relation to vignettes depicting bullying (H1a) and when the target of bullying is autistic (H1b) and also to explore responses to different bullying violations, 2 (school exposure: centre vs non-centre) × 2 (target type: NT vs ASD) × 2 (bullying violation: verbal vs social exclusion) ANOVAs of change scores were conducted, separately for each of the DVs. Given the significant main effect of personal contact (reported below), this was included as a factor to keep this variable constant when we explored differences by school exposure. Table 1 displays the mean values and standard deviations of change scores. (Mean values and standard deviations of Time 1 and Time 2 scores can be found in Supplemental Material S2.)

**Table 1.** Change in responses to vignettes by school exposure.

	Centre school (*N* = 426)				Non-centre school (*N* = 349)			
	ASD target (*N* = 200)		NT target (*N* = 226)		ASD target (*N* = 155)		NT target (*N* = 194)	
	Verbal bullying (*N* = 107)	Social exclusion (*N* = 93)	Verbal bullying (*N* = 120)	Social exclusion (*N* = 106)	Verbal bullying (*N* = 79)	Social exclusion (*N* = 76)	Verbal bullying (*N* = 98)	Social exclusion (*N* = 96)
Change in judgement	*x̅* = 0.00 *sd* = 0.54	*x̅* = 0.04 *sd* = 0.65	*x̅* = 0.01 *sd* = 0.47	*x̅* = 0.02 *sd* = 0.66	*x̅* = 0.00 *sd* = 0.51	*x̅* = 0.16 *sd* = 0.69	*x̅* = 0.01 *sd* = 0.55	*x̅* = –0.10 *sd* = 0.73
Change in emotions	*x̅* = 0.02 *sd* = 0.38	*x̅* = 0.02 *sd* = 0.53	*x̅* = 0.01 *sd* = 0.50	*x̅* = 0.05 *sd* = 0.54	*x̅* = –0.10 *sd* = 0.46	*x̅* = 0.07 *sd* = 0.52	*x̅* = –0.03 *sd* = 0.43	*x̅* = –0.15 *sd* = 0.60
Change in intended behaviour	*x̅* = –0.01 *sd* = 0.52	*x̅* = –0.10 *sd* = 0.60	*x̅* = –0.034 *sd* = 0.60	*x̅* = –0.13 *sd* = 0.55	*x̅* = –0.10 *sd* = 0.43	*x̅* = –0.05 *sd* = 0.66	*x̅* = –0.05 *sd* = 0.46	*x̅* = –0.13 *sd* = 0.67

ASD: autistic spectrum disorder; NT: neurotypical.

**Figure 1.**
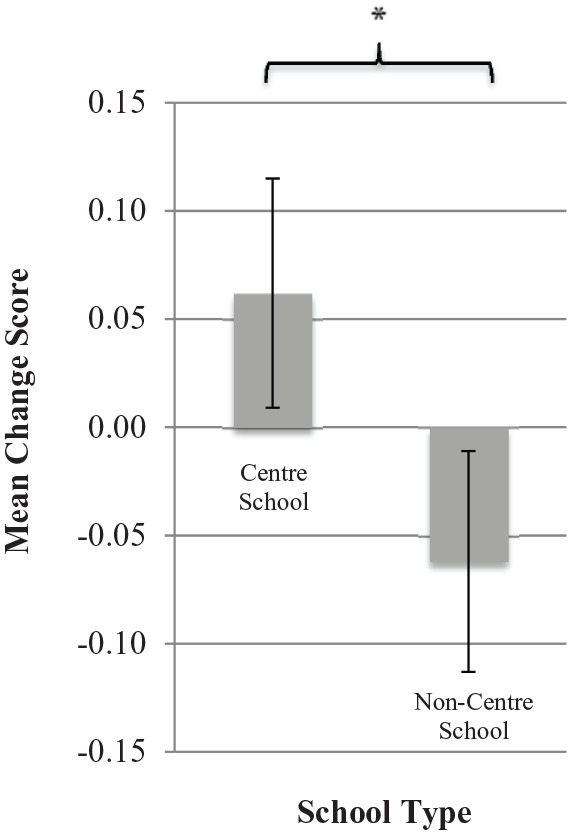
Change in prosocial emotions towards bullying by school type. Error bars represent standard error. *p < 0.05.

There were no significant differences between centre schools and non-centre schools for judgements and intended behaviours. However, consistent with H1a, results revealed a significant main effect of school exposure on change in emotions, *F*(1, 638) = 5.47, *p* = 0.02, $\eta_{p}^{2}$ = 0.01 (Figure 1).

These results indicate that participants from non-centre schools showed a decrease in prosocial emotions towards bullying (T1: *M* = 4.31, *SD* = 0.50; T2: *M* = 4.28, *SD* = 0.53) in comparison to participants from centre schools who showed an increase (T1: *M* = 4.29, *SD* = 0.47; T2: *M* = 4.31, *SD* = 0.50).

**Figure 2.**
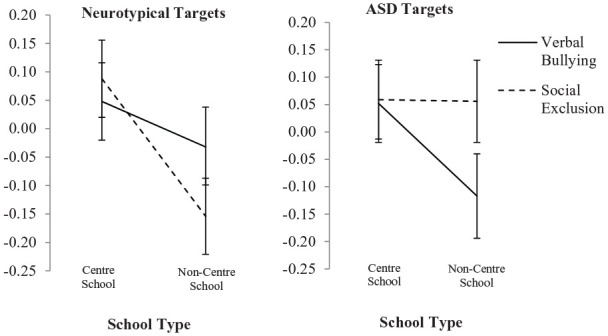
Change in prosocial emotions towards bullying by school type (three-way interaction). Error bars represent standard error.

Results also showed a three-way interaction of school exposure × target × bullying violation, *F*(1, 638) = 4.31, *p* = 0.04, $\eta_{p}^{2}$ (Figure 2), indicating that while verbal bullying produced similar changes in prosocial emotions between ASD and NT targets, social exclusion presented a different picture, whereby participants in non-centre schools showed a decrease in prosocial emotions towards NTs subject to social exclusion (T1: *M* = 4.30, *SD* = 0.44; T2: *M* = 4.19, *SD* = 0.54), but an increase in prosocial emotions towards social exclusion when the target was autistic (T1: *M* = 4.26, *SD* = 0.56; T2: *M* = 4.34, *SD* = 0.48) (matching participants from centre schools).

**Figure 3.**
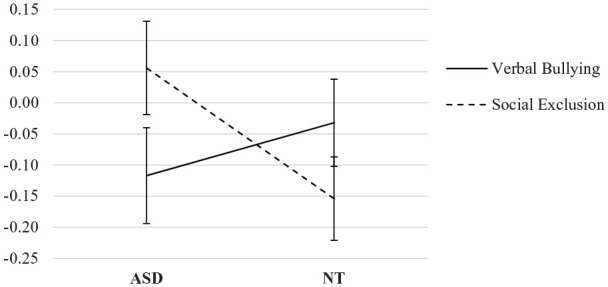
Change in prosocial emotions towards bullying of ASD targets and NT targets in non-centre schools. Error bars represent standard error.

To explore this significant interaction further, 2-way target × bullying violation ANOVAs were conducted for each type of school exposure (centre and non-centre). This revealed a significant interaction for non-centre schools of target × bullying violation, *F*(1, 286) = 6.145, *p* = 0.014, $\eta_{p}^{2}$ = 0.021, indicating that in non-centre schools, verbal bullying resulted in a greater decrease in prosocial emotions for ASD targets (*M* (change score) = −0.12, *SD* = 0.61) than for NT targets (*M* (change score) = −0.03, *SD* = 0.65), but the opposite effect was true for social exclusion, which resulted in a decrease in prosocial emotions for NT targets (*M* (change score) = −0.15, *SD* = 0.61), but an increase for ASD targets (*M* (change score) = 0.06, *SD* = 0.59) (Figure 3).

#### DV 4: attitudes to autism

To test the hypothesis that attending a school with a centre would lead to a greater increase in positive attitudes towards people on the autistic spectrum (H1c), an ANOVA of the change scores was conducted, also as a custom model where personal contact was held constant. There was no significant difference between centre schools and non-centre schools.

### The impact of change in personal contact with autistic people

ANOVAs were conducted to measure change in attitudes according to their change in personal contact with autistic people (decrease, no change or increase, as described above).

#### DVs 1, 2 and 3: judgements, emotions and intended behaviours

To test the hypotheses that an increase in personal contact with autistic people would lead to a greater increase in prosocial judgements, emotions and intended behaviours in relation to vignettes depicting bullying (H2a) and when the target of bullying is autistic (H2b) and also to explore responses to different bullying violations, 3 (personal contact: decrease; no change; increase) × 2 (target type: NT vs ASD) × 2 (bullying violation: verbal bullying vs social exclusion) ANOVAs of change scores were conducted separately for each of the DVs. Table 2 displays the mean values and standard deviations.

**Table 2.** Change in responses to vignettes by change in personal contact.

	Decrease (*N* = 78)				No change (*N* = 488)				Increase (*N* = 124)			
	ASD target (*N* = 35)		NT target (*N* = 43)		ASD target (*N* = 229)		NT target (*N* = 259)		ASD target (*N* = 49)		NT target (*N* = 75)	
	Verbal bullying (*N* = 15)	Social exclusion (*N* = 20)	Verbal bullying (*N* = 19)	Social exclusion (*N* = 24)	Verbal bullying (*N* = 122)	Social exclusion (*N* = 107)	Verbal bullying (*N* = 138)	Social exclusion (*N* = 121)	Verbal bullying (*N* = 25)	Social exclusion (*N* = 24)	Verbal bullying (*N* = 39)	Social exclusion (*N* = 36)
Change in judgement	*x̅* = –0.26 *sd* = 0.60	*x̅* = 0.04 *sd* = 0.83	*x̅* = –0.07 *sd* = 0.38	*x̅* = –0.30 *sd* = 0.94	*x̅* = 0.05 *sd* = 0.53	*x̅* = 0.07 *sd* = 0.61	*x̅* = 0.05 *sd* = 0.49	*x̅* = –0.00 *sd* = 0.67	*x̅* = –0.06 *sd* = 0.43	*x̅* = 0.26 *sd* = 0.76	*x̅* = –0.07 *sd* = 0.62	*x̅* = 0.04 *sd* = 0.56
Change in emotions	*x̅* = –0.14 *sd* = 0.43	*x̅* = 0.06 *sd* = 0.59	*x̅* = 0.10 *sd* = 0.38	*x̅* = –0.18 *sd* = 0.74	*x̅* = 0.00 *sd* = 0.42	*x̅* = 0.02 *sd* = 0.49	*x̅* = –0.02 *sd* = 0.43	*x̅* = –0.03 *sd* = 0.54	*x̅* = –0.13 *sd* = 0.41	*x̅* = 0.13 *sd* = 0.65	*x̅* = –0.01 *sd* = 0.63	*x̅* = –0.01 *sd* = 0.58
Change in intended behaviour	*x̅* = –0.09 *sd* = 0.54	*x̅* = –0.14 *sd* = 0.57	*x̅* = –0.12 *sd* = 0.48	*x̅* = –0.11 *sd* = 0.71	*x̅* = –0.01 *sd* = 0.47	*x̅* = –0.07 *sd* = 0.58	*x̅* = –0.04 *sd* = 0.56	*x̅* = –0.16 *sd* = 0.52	*x̅* = –0.20 *sd* = 0.55	*x̅* = 0.23 *sd* = 0.83	*x̅* = –0.01 *sd* = 0.50	*x̅* = –0.06 *sd* = 0.79

ASD: autistic spectrum disorder; NT: neurotypical.

Results revealed a significant main effect of change in personal contact for change in judgements, *F*(2, 641) = 3.19, *p* = 0.04, $\eta_{p}^{2}$ = 0.01. However, due to lack of homogeneity of variance for this variable, and unequal sample sizes, a Kruskal–Wallis test was conducted and this showed no significant difference in change of judgements according to change in personal contact, (χ^2^ = 4.12, *p* = 0.13).

#### DV 4: attitudes to autism

**Figure 4.**
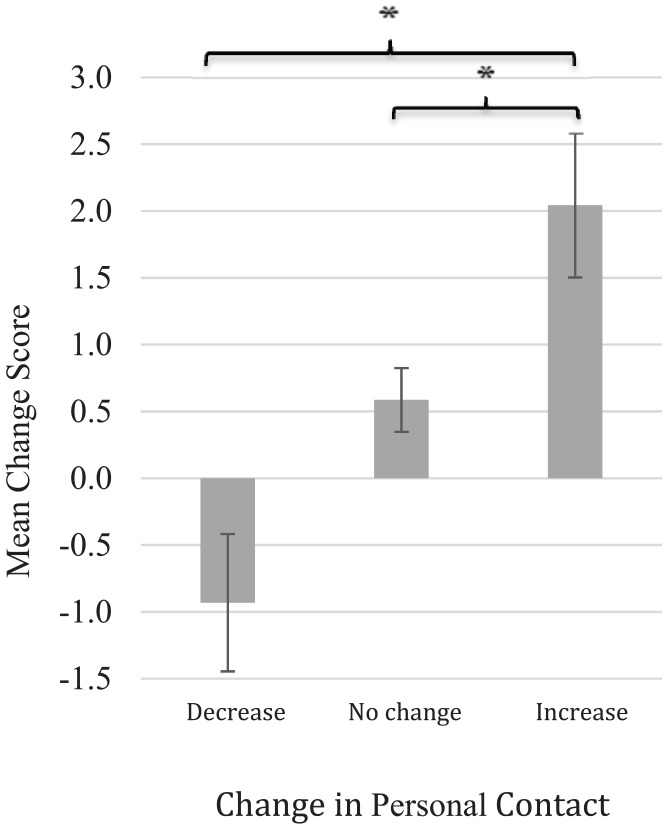
Change in attitudes towards autistic people according to change in personal contact. Error bars represent standard error. *p < 0.05.

To test the hypothesis that an increase in personal contact with autistic people would lead to a greater increase in positive attitudes towards autistic people (H2c), an ANOVA comparing change in attitudes towards autistic people according to change in personal contact was conducted. Consistent with H2c, this revealed a significant effect of change in personal contact, *F*(2, 653) = 7.771, *p* < 0.001, $\eta_{p}^{2}$ = 0.023. Post hoc comparisons indicated that the mean change score for the ‘Increase’ group (*M* = 2.042, *SD* = 5.851) was significantly higher than the mean change score for the ‘Decrease’ group (*M* = −0.932, *SD* = 4.421), *p* < 0.001. The mean change score for the ‘Increase’ group was also significantly higher than the mean change score for ‘no change’ group (*M* *=* 0.586, *SD* = 5.164), *p* = 0.019 (Figure 4). This indicates that participants who increased their personal contact with people on the autistic spectrum reported a greater increase in positive attitudes towards them (T1: *M* = 22.02, *SD* = 4.86; T2: *M* = 24.05, *SD* = 4.60) than participants with no change in personal contact (T1: *M* = 22.20, *SD* = 5.02; T2: *M* = 22.84, *SD* = 5.10) and also participants who decreased their personal contact (T1: *M* = 23.44, *SD* = 4.54; T2: *M* = 22.67, *SD* = 4.63).

## Discussion

The opening of purpose-built specialist centres in three mainstream schools in England provided a unique opportunity to examine the impact of school exposure and personal contact with autistic peers on NT pupils’ evaluations of the bullying of people on the autistic spectrum. In line with this, this study investigated the impact of contrasting school settings and personal contact on judgements, emotions, intended behaviours and attitudes towards bullying and autism. A vignette was used as a means to measure the key outcome variables and to assess the additional impact of different targets of bullying and different bullying violations.

In partial support of H1a, results showed that pupils in centre schools showed a greater increase in prosocial emotions (but not prosocial judgements or intended behaviours) towards hypothetical bullying scenarios. They did not however support H1b or H1c (that pupils in centre schools would show greater prosocial responses when targets of bullying are autistic or more positive attitudes towards autism).

Second, an interaction showed that for participants in non-centre schools, while there was a decrease in prosocial emotions towards social exclusion when the target was NT, they reported an increase in prosocial emotions when the target was autistic (matching participants from centre schools).

In support of H2c, pupils who increased their personal contact with people on the autistic spectrum showed a greater increase in positive attitudes towards them than pupils with no change or a decrease in personal contact. However, findings did not support H2a or H2b (that pupils who increase their personal contact would show greater prosocial responses towards bullying or when targets of bullying are autistic). These findings are explained below.

Pupils from centre schools showed an increase in prosocial emotions towards hypothetical bullying scenarios. Schools that emphasize moral responsibility and a sense of community have been found to influence inclusive and exclusive peer group norms (Colby & Kohlberg, 1987; Killen & Rutland, 2011). While no differences were found in pupil’s judgements or intended behaviours, one interpretation of the finding from this study is that the increased emphasis placed on establishing an inclusive culture in centre schools may contribute to an increase in prosocial emotional reactions to bullying, irrespective of the bullying violation or target (although it should be noted that the effect size was small).

The second finding in relation to school exposure showed that that for participants in non-centre schools, while there was a decrease in prosocial emotions in response to verbal bullying and social exclusion when the target was NT, interestingly they reported an increase in prosocial emotions when the target was autistic. Verbal bullying is reported to be the most common form of bullying (Ahmed & Smith, 1994; Björkqvist et al., 1992; Boulton et al., 2002) so a possible explanation for the decrease in prosocial emotions for verbal bullying in non-centre schools is that it is accepted as a social norm in these schools, and hence more likely to be tolerated in order to preserve group functioning. In contrast, while previous research suggests that social exclusion is least likely to be perceived as bullying (Boulton et al., 2002), it may be the case that perceptions are different when the target has disabilities/SEN. In contrast with H1b (that pupils in centre schools would show greater prosocial responses when targets of bullying are autistic), it may be that social exclusion of an autistic peer may raise more moral considerations for NT children, regardless of school exposure. Further research is needed to investigate this hypothesis further.

In terms of the impact of personal contact, pupils who increased their personal contact with people on the autistic spectrum showed more positive attitudes towards them. Accepting that NT and ASD pupils are often perceived as representing different groups, this finding can be explained by the contact hypothesis (Allport, 1954), which states that contact between groups can disconfirm stereotypes and instil more positive attitudes, beliefs and behaviours. Much research based on the contact hypothesis focuses on ethnic groups, and reports less bias against different ethnic groups following high-quality cross-group friendship (Aboud et al., 2003; Feddes et al., 2009). Fewer studies have explored the effects of contact with children with disabilities, but from the limited studies, again findings indicate that contact leads to more liking of and peers with disabilities and condemnation of their exclusion (Gasser et al., 2013; Grütter et al., 2017; Maras & Brown, 1996). In contrast to many cross-sectional or correlational studies where contact is reported at one time point, this study was longitudinal, collecting responses at two time points and may therefore suggest a tentative causal link between personal contact and subsequent attitudes. It is important to note however that while this study measured changes in quantity of time spent with people on the autistic spectrum, this cannot signify quality of time spent. The success of inter-group contact is reliant on positive interaction. Research has started to explore quality of friendship for autistic people (Kasari et al., 2011; Petrina et al., 2014), but future research might also explore friendships between autistic and NT children to give a more complete understanding of friendship quality in these inter-group relationships.

Finally, comparing results of the two types of contact reveals a difference in the quality of attitudes, whereby personal contact led to greater changes in cognitive attitudes while differences in school exposure highlighted differences in emotional responses to bullying. For personal contact, not only were there significant differences in cognitive attitudes towards autistic people, but also in judgements towards the vignette (*p* = 0.04) although this violated the assumption of homogeneity of variance. It does however point to a possible pattern in differences in the more cognitive attitudes in response to personal contact.

In contrast, according to the different types of school exposure, differences were found in emotion response, supporting the findings of Gasser et al. (2013) that children from inclusive classrooms express more moral emotions towards social exclusion and a greater likelihood to include children with disabilities. In this study, the presence of significant differences in emotional responses and absence of differences according to their judgements/intended behaviours point to the possibility that children in centre schools feel that bullying is not fair, and respond to this in an emotional sense, but may not show increased prosocial cognitive responses or behavioural intentions due to the importance of protecting their group and its social conventions, in accordance with social domain theory (Turiel, 2008).

Very little research examines the emotions attributed to participants in bullying/excluding scenarios. One such study by Malti et al. (2012) asked 12- and 15-year-old Swiss and non-Swiss adolescents to judge exclusion based on nationality and found that Swiss participants (the ingroup) attributed fewer positive emotions to excluders. The findings in the current study showed how participants similarly attributed emotions to themselves as hypothetical bystanders, and for participants in centre schools indicated a greater increase in anger, pity, sadness and shame and/or decrease in pride, excitement, amusement and satisfaction in response to the bullying, irrespective of target type.

These findings highlight the importance of contact, both at a personal level and through attending a school with an inclusive autism provision. While the provision type may have affected prosocial emotions in response to bullying, personal contact is vital for positive cognitive attitudes, and in support of Pettigrew (1998) simply attending a ‘mixed school’ is not enough. If there are differences in the way information is processed according to type of contact, this should inform types of interventions that may be best suited to children in particular contexts and stages of development.

These findings may have implications for education authorities in their consideration of the physical and social provision that not only meets the needs of autistic children, but also supports inclusive beliefs, and sense of community that may intensify NT children’s emotional reactions to bullying. Furthermore, while thorough consideration of the physical and social school climate is important, contact-based interventions are also vital for improvements to attitudes towards people on the autistic spectrum. Positive contact can disconfirm stereotypes and increase liking between peers, as shown in this study.

### Limitations

This study had a number of limitations: (a) given that the centres had only recently opened, there is a possibility that an inclusive school ethos had not yet been established to the extent to which more significant differences could be measured compared to control schools (including those of H1b and H1; c) Unfortunately, long-term durability of these findings could not be tested, due to time constraints; (b) in order to control for factors of stigma in the vignettes, the NT target of bullying was described as being self-conscious about their weight. Therefore, vignettes were not matched in length or the number of attributes used to describe each target of bullying. In contrast with the NT target, the attributes of the autistic target were not value neutral (using words such as ‘anxious’ and ‘angry’) and could therefore have been conceived as potentially more stigmatizing than the NT target and may partially explain why some hypotheses were not supported, and for those that were, why the effect sizes were small in magnitude; (c) Also in relation to vignette design, it cannot be guaranteed that hypothetical behaviour will exactly reflect their actual behaviour, but evidence shows that the two correspond (Langley et al., 1991; Lunza, 1990; Murphy et al., 1986); (d) due to time restrictions participants could not be shown all four vignettes, which might have affected the power of the study; (e) the study may be limited by a response system that could stimulate socially desirable answers, particularly in schools that promote an inclusive ethos. Furthermore, NT people can believe that they are overly helpful to autistic people, when their real-life behaviours do not reflect this (Heasman & Gillespie, 2019); (f) there may be analytical limitations due to the multiple comparisons associated with a three-way ANOVA. However, while some advocate that alpha levels should therefore be adjusted to account for multiple comparisons (Cramer et al., 2016), others believe this to cause greater problems since doing so increases the potential for type 2 errors (Perneger, 1998).

In conclusion, this study showed that that while personal contact with autistic people facilitated a greater increase in positive attitudes towards people on the autistic spectrum, attending a school with a specialist centre for autism facilitated a greater increase in prosocial emotional responses to bullying. It could be speculated that this model of provision leads pupils to be more accepting of difference and less tolerant of bullying. The findings also show that positive contact is vital for improvements to attitudes towards autism.

## Supplemental Material

Supplementary_Material_AUT-19-0401.R3 – Supplemental material for The effect of school exposure and personal contact on attitudes towards bullying and autism in schools: A cohort study with a control group Click here for additional data file. Supplemental material, Supplementary_Material_AUT-19-0401.R3 for The effect of school exposure and personal contact on attitudes towards bullying and autism in schools: A cohort study with a control group by Anna Cook, Jane Ogden and Naomi Winstone in Autism
