# Supplementary material for: The effect of school exposure and personal contact on attitudes towards bullying and autism in schools: A cohort study with a control group
Source: Autism. 2020 Jul 15;24(8):2178–89. doi: 10.1177/1362361320937088 (PMC7549291; doi:10.1177/1362361320937088)
Supplement: Supplementary_Material_AUT-19-0401.R3 – Supplemental material for The effect of school exposure and personal contact on attitudes towards bullying and autism in schools: A cohort study with a control group [file Supplementary_Material_AUT-19-0401.R3.pdf]

Supplemental material:
The effect of school exposure and personal contact on attitudes towards bullying and autism in schools: A cohort study with a control group

S1: Vignette Types

[Bulling target: NT]	[Bulling target: ASD]
Emily/Jack is a girl/boy in your year group. You don't know her/him well, but know that she/he is self-conscious about her/his weight. One day…	*Emily/Jack is a girl/boy in your year group. You don't know her/him well, but have been told that she/he has autism – a brain condition that causes her/him to have difficulties communicating with other people and to get anxious and even angry when things change unexpectedly or when there is lots of noise. One day…*
[Verbal bullying]<br><br>*…you hear Amy/William - another girl/boy from your form group - call her/him 'freak'. This is not the first time it's happened. On a previous occasion nasty things had been posted about Emily/Jack on social media. Emily/Jack tries to ignore the remark, but goes to sit on her/his own looking sad.*	
[Social exclusion]<br><br>*…Emily/Jack walks up to you and some friends from your form and asks if she/he can join in your conversation. Amy/William - one of the girls/boys in your group says "no, we're having a private conversation" and then turns her/his back on Emily/Jack to indicate that she/he should leave. This is not the first time it's happened.*	

S2: Responses to Vignettes by School Exposure and Target Type

	Centre School N=426				Non-Centre School N=349			
	ASD Target N=200		NT Target N=226		ASD Target N=155		NT Target N=194	
	Time 1	Time 2	Time 1	Time 2	Time 1	Time 2	Time 1	Time 2
Judgments	$\bar{x}$ = 4.38<br>*sd* = 0.50	$\bar{x}$ = 4.40<br>*sd* = 0.44	$\bar{x}$ = 4.26<br>*sd* = 0.57	$\bar{x}$ = 4.27<br>*sd* = 0.54	$\bar{x}$ = 4.32<br>*sd* = 0.60	$\bar{x}$ = 4.41<br>*sd* = 0.53	$\bar{x}$ = 4.28<br>*sd* = 0.54	$\bar{x}$ = 4.26<br>*sd* = 0.60
Emotions	$\bar{x}$ = 4.34<br>*sd* = 0.45	$\bar{x}$ = 4.36<br>*sd* = 0.46	$\bar{x}$ = 4.25<br>*sd* = 0.49	$\bar{x}$ = 4.27<br>*sd* = 0.48	$\bar{x}$ = 4.32<br>*sd* = 0.53	$\bar{x}$ = 4.33<br>*sd* = 0.50	$\bar{x}$ = 4.30<br>*sd* = 0.48	$\bar{x}$ = 4.23<br>*sd* = 0.54
Intended behaviours	$\bar{x}$ = 4.22<br>*sd* = 0.51	$\bar{x}$ = 4.14<br>*sd* = 0.59	$\bar{x}$ = 4.20<br>*sd* = 0.54	$\bar{x}$ = 4.13<br>*sd* = 0.58	$\bar{x}$ = 4.21<br>*sd* = 0.58	$\bar{x}$ = 4.19<br>*sd* = 0.61	$\bar{x}$ = 4.20<br>*sd* = 0.60	$\bar{x}$ = 4.13<br>*sd* = 0.62
